# An *in vitro* Propagation of *Aspilia africana* (Pers.) C. D. Adams, and Evaluation of Its Anatomy and Physiology of Acclimatized Plants

**DOI:** 10.3389/fpls.2021.704896

**Published:** 2021-07-29

**Authors:** Denis Okello, Sungyu Yang, Richard Komakech, Endang Rahmat, Yuseong Chung, Roggers Gang, Yong-Goo Kim, Francis Omujal, Youngmin Kang

**Affiliations:** ^1^Herbal Medicine Resources Research Center, Korea Institute of Oriental Medicine (KIOM), Naju-si, South Korea; ^2^Korean Convergence Medicine Major, University of Science and Technology (UST), Daejeon, South Korea; ^3^Natural Chemotherapeutics Research Institute (NCRI), Ministry of Health, Kampala, Uganda; ^4^National Semi-Arid Resources Research Institute (NaSARRI), Soroti, Uganda; ^5^Biological Resource Center, Korea Research Institute of Bioscience and Biotechnology (KRIBB), Jeongeup-si, South Korea

**Keywords:** anatomy, *Aspilia africana*, FT-NIR, *in vitro* propagation, micropropagation, nodal segments, physiology

## Abstract

*Aspilia africana* (Pers.) C. D. Adams is an important medicinal plant, that has been used as traditional medicine in many African countries for the treatment of various health problems, including inflammatory conditions, osteoporosis, tuberculosis, cough, measles, diabetes, diarrhea, malaria, and wounds. We developed an efficient and reproducible protocol for *in vitro* regeneration of *A. africana* from nodes. We assessed the effects of plant tissue culture media on *A. africana* growth, cytokinins for *in vitro* shoot regeneration and proliferation, and auxins for the rooting of regenerated shoots. Furthermore, chlorophyll content, photosynthetic rates, anatomy (leaves, stems, and roots), and Fourier transform near-infrared (FT-NIR) spectra (leaves, stems, and roots) of the *in vitro* regenerated and maternal *A. africana* plants were compared. Murashige and Skoog media, containing vitamins fortified with benzylaminopurine (BA, 1.0 mg/l), regenerated the highest number of shoots (13.0 ± 0.424) from *A. africana* nodal segments. 1-naphthaleneacetic acid (NAA, 0.1 mg/l) produced up to 13.10 ± 0.873 roots, 136.35 ± 4.316 mm length, and was the most efficient for rooting. During acclimatization, the *in vitro* regenerated *A. africana* plants had a survival rate of 95.7%, displaying normal morphology and growth features. *In vitro* regenerated and mother *A. africana* plants had similar chlorophyll contents, photosynthetic rates, stem and root anatomies, and FT-NIR spectra of the leaf, stem, and roots. The established regeneration protocol could be used for large-scale multiplication of the plant within a short time, thus substantially contributing to its rapid propagation and germplasm preservation, in addition to providing a basis for the domestication of this useful, high-value medicinal plant.

## Introduction

*Aspilia africana* is one of the most valuable medicinal plants in the wild and is widely used in communities where it occurs (Okoli et al., 2007; Eweka and Eweka, 2008; Okello and Kang, 2019; Niyonizigiye et al., 2020). In Uganda, *A. africana* is 1 of the top 17 most effective herbal plants used for the treatment of malaria (Okello and Kang, 2019) and in Cameroon, it is the most commonly used plant for treating wounds (Simbo, 2010). *Aspilia africana* is indigenous to East Africa, although it occurs in forest zones in all regions of tropical Africa and the savanna in countries such as Tanzania, Uganda, Congo, Ghana, Liberia, Sudan, Central African Republic, Mali, Ethiopia, Cameroon, Burkina Faso, Nigeria, Senegal, and Niger (Komakech et al., 2019; Okello et al., 2020).

*Aspilia africana* (Pers.) C. D. Adams, commonly referred to as the wild sunflower or the hemorrhage plant, is a plant species in the Asteraceae family that has been used for generations in a number of African countries to treat a range of health conditions (Ajeigbe et al., 2014; Okello et al., 2020). This medicinal plant is used in the treatment of inflammatory conditions, osteoporosis, stomach ache, tuberculosis, cough, measles, diabetes, rheumatic pains, ear infections, gastric ulcers, diarrhea, malaria, sores, bee, scorpion and wasp stings, febrile headaches, wounds, gonorrhea, and it serves as a contraceptive as well (Okoli et al., 2007; Eweka and Eweka, 2008; Komakech et al., 2019; Okello and Kang, 2019). A recent study pointed out that *A. africana* has anticancer activity, with a remarkable effect on adenocarcinoma gastric cell lines (AGS) (Niyonizigiye et al., 2020). *Aspilia africana* is rich in a number of secondary metabolites, including flavonoids (such as quercetin), phenolic compounds (including gallic acid and chlorogenic acid) (Niyonizigiye et al., 2020), terpenes (including *beta*-Caryophyllene, *alpha*, and *beta*-pinene and phytol; Komakech et al., 2019; Okello et al., 2020), saponins, and tannins accounting for its broad antimicrobial and biological activities, including anti-inflammatory, hemostatic, oxytocic, gastro-protective, anti-ulcer, wound healing, anti-cancer, anti-hypertensive, and anti-diabetic potential (Okoli et al., 2007; Ajeigbe et al., 2014; Komakech et al., 2019).

There is no literature to date for the *in vitro* propagation of *A. africana*, although it is not only an important medicinal plant of pharmaceutical and cosmetic interests but is also used as feed for domestic animals, such as cattle, goats, rabbits, and sheep, in Africa (Ahamefule et al., 2006; Okoli et al., 2007; Oko et al., 2017; Komakech et al., 2019). In our earlier investigation on seed germination of *A. africana* in different soil types, the plant had very low final percentage seed germination (ranging from 11.67 ± 0.882% to 15.67 ± 1.202%), which did not vary significantly among the soil types (data not yet published, manuscript under review). Due to limitations arising from conventional plant propagation methods through seeds (such as viability and increased chances of seed borne diseases) and by vegetative means (such as increased risks of disease transmission to propagules from mother stock and production of a limited number of plants), there is need for an alternate method for large-scale propagation of this plant. Micropropagation plays a crucial role not only in meeting conservation needs but also in supplying quality medicinal plant stock to meet growing pharmaceutical demand (Teixeira da Silva et al., 2019; Kher et al., 2020; Komakech et al., 2020). *In vitro* propagation is known to be more effective for the rapid multiplication of plants than the conventional propagation (Das et al., 2020). The current study aimed to develop an effective *in vitro* propagation protocol for *A. africana* from nodal explants. We examined the effects of different plant tissue culture media on the growth of *A. africana* and tested the effects of plant growth regulators (PGRs) on optimum shoot proliferation and rooting of regenerated *A. africana* plants. Owing to its high medicinal and economic value, developing a micropropagation technique for *A. africana* would not only provide elite clones for its pharmaceutical use and promote its rapid propagation and germplasm conservation but would also promote its domestication and aid in the preservation of wild populations.

*In vitro* propagation may result in physiological and anatomical features intrinsic to the media culture environment (Manokari et al.,2021a,b). Morpho-anatomical, molecular, and biochemical attributes have been used to monitor the stability and integrity of *in vitro* regenerated plants (Sonibare and Adeniran, 2014). Anatomical features are very important and play key roles in plant identification (Manokari et al.,2021a,b). We examined and compared the detailed anatomical features of the leaves, stems, and roots of *in vitro* regenerated *A. africana* plants and their maternal stocks. Furthermore, Soil Plant Analysis Development (SPAD), FluorPen FP110 series, and Fourier transform near-infrared (FT-NIR) spectrometry were used to compare chlorophyll pigment contents, photosynthetic rates, and chemical compositions of the *in vitro* regenerated *A. africana* and the mother plants.

## Materials and Methods

### Seed Collection and Explant Preparation

**FIGURE 1:**
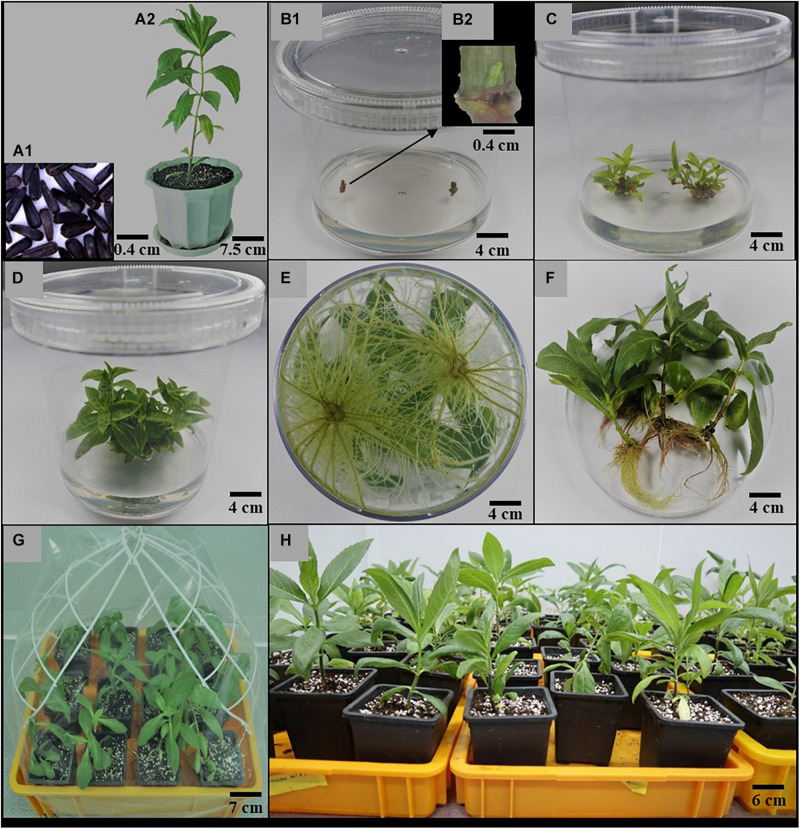
Summary of *in vitro* Propagation of *A. africana.* **(A1)** Seeds of *A. africana*. **(A2)** Four-month-old *A. africana* maternal plant. **(B1)** Nodal segments inoculated in MS medium supplemented with BA (1.0 mg/l). **(B2)** Morphology of inoculated nodal segment showing the shoot bud. **(C)** Proliferated shoots in same medium [in **(B)**] after 3 weeks. **(D)** Proliferated shoots in same medium as in **(B)** after 6 weeks. **(E)** Fully developed roots in MS medium supplemented with NAA (0.1 mg/l) at 4 weeks. **(F)** Plantlets removed from medium and roots washed. **(G)** Regenerated *A. africana* plantlets transferred to soil and covered with polythene bags to maintain moisture **(H)**. acclimatized potted plants.

Mature and dry seeds (Figure 1A1) of *A. africana*, randomly collected from at least 50 healthy plants from Pece in Gulu district, Uganda, East Africa, were provided by the Natural Chemotherapeutics Research Institute, Uganda. A voucher specimen (number KYM-KIOM-2021-1) was deposited at the Korean Herbarium of Standard Herbal Resources (Index Herbarium code: KIOM) at the Korea Institute of Oriental Medicine (KIOM), Herbal Medicine Resources Research Center, Republic of South Korea. The seeds were planted in plastic pots (22 cm in diameter), containing a mixture of autoclaved horticulture soil and perlite in a 2:1 ratio, and maintained within growth chambers (JSCC-460CP model, Js Research Inc. 40-1, Gumsang-dong, Gongju, South Korea) under a 16-h photoperiod (33.73 μmol/m^2^/s light intensity provided by cool white fluorescent tubes) at a temperature of approximately 25°C and relative humidity of 80%. After a 4-month growth period of the *A. africana* plants (Figure 1A2), shoot tips (50–70 mm) were collected and thoroughly washed under running tap water for approximately 10 min and transferred to a laminar flow clean bench. The shoot tips of *A. africana* were washed again with double-distilled autoclaved water and then surface-sterilized in 100% (v/v) and 70% (v/v) ethanol for 30 s each, followed by 2% (v/w) sodium hypochlorite for 2 min, and rinsed thrice with sterile water. The sterilized *A. africana* shoot apices were further cut into smaller pieces (25–40 mm) with sterile scalpels to remove cut end surfaces that were in direct contact with the sterilizing agents. These shoot tips were used to investigate the effects of different plant culture media on the growth of *A. africana.* After an additional 6 weeks, *A. africana* shoots were collected from the soil-based plants, cut into nodal segments of about 30 mm in length, and then surface sterilized in the same way as the shoot tips. The sterilized *A. africana* nodal segments were then further cut into smaller pieces, of approximately 20 mm, with sterile scalpels to remove their end surfaces that were in direct contact with the sterilizing agents. Stem nodal segments were used for the *in vitro* regeneration experiments.

### Effects of Culture Media on the Growth of *A. africana* Shoots

The effect of six different basal media on the shoot growth of *A. africana* was investigated. Two excised shoot apices (25–40 mm) were carefully inoculated into 100 ml of each of the six gelled media [Woody Plant Medium (WPM), Murashige and Skoog (MS), Linsmaier and Skoog (LS), De Greef and Jacobs (DJ), Nitsch medium (NM), and Quoirin and Lepoivre (QL)] in a polystyrene culture vessel (125 × 110 mm, Gaooze 1011C culture vessel, Gyeonggi-do, South Korea). Before inoculation, the initial length, fresh weight, and initial number of leaves were determined for each shoot apex. Fifteen replicates (two explants per culture vessel and fifteen culture vessels for each treatment) were made and repeated thrice. All cultures in this experiment were maintained under a 16-h photoperiod (with a light intensity of 33.73 μmol/(m^2^/s) provided by cool white fluorescent tubes) and a relative humidity of 80%. All basal media used in the study contained vitamins and were supplemented with 3% sucrose at a pH of 5.8, solidified with 3 g/l gelrite, and autoclaved at 121°C for approximately 20 min. After a culture period of 6 weeks, the final lengths, fresh weights, and final number of leaves were determined for all the *A. africana* shoot apices in the media for subsequent calculations as follows:

$$\mathrm{Percentageincreaseinshootlength}\;=\frac{F\,i\,n\,a\,l\,s\,h\,o\,o\,t\,l\,e\,n\,g\,t\,h-I\,n\,i\,t\,i\,a\,l\,s\,h\,o\,o\,t\,l\,e\,n\,g\,t\,h}{I\,n\,i\,t\,i\,a\,l\,s\,h\,o\,o\,t\,l\,e\,n\,g\,t\,h}\times 100;$$

$$\mathrm{Percentageincreaseinfreshweight}\;=\frac{F\,i\,n\,a\,l\,f\,r\,e\,s\,h\,w\,e\,i\,g\,h\,t-I\,n\,i\,t\,i\,a\,l\,f\,r\,e\,s\,h\,w\,e\,i\,g\,h\,t}{I\,n\,i\,t\,i\,a\,l\,f\,r\,e\,s\,h\,w\,e\,i\,g\,h\,t}\times 100$$

$$\mathrm{Percentageincreaseinnumberofleaves}\;=\frac{F\,i\,n\,a\,l\,n\,u\,m\,b\,e\,r\,o\,f\,l\,e\,a\,v\,e\,s-I\,n\,i\,t\,i\,a\,l\,n\,u\,m\,b\,e\,r\,o\,f\,l\,e\,a\,v\,e\,s}{I\,n\,i\,t\,i\,a\,l\,n\,u\,m\,b\,e\,r\,o\,f\,l\,e\,a\,v\,e\,s}\times 100.$$

The basal medium with visibly healthy plants and the highest average value for all three *A. africana* growth parameters used, MS, was considered overall best for growth and was used in subsequent experiments.

All media, gelling agent and PGRs used in this study were purchased from Duchefa Biochemie B. V., Haarlem, Netherlands and all were of American Chemical Society (ACS) grade.

### Effects of Cytokinins on Shoot Proliferation of *A. africana*

The stem nodal segments (approximately 20 mm in length) were cultured in MS basal medium supplemented with five different cytokinins, kinetin (Kn), 6-Benzylaminopurine (BA), isopentenyl adenine (2iP), zeatin, and thidiazuron (TDZ) at different concentrations (0.1, 0.5, 1.0, 1.5, and 2.0 mg/L). For each treatment, two nodal segments were inoculated in 100 ml of each medium in a culture vessel (125 × 110 mm) (Figures 1B1,B2) and replicated 20 times. After 6 weeks, the number of shoots formed from each nodal segment (Figures 1C,D) was counted and recorded.

### Effects of Auxins on the Initiation and Growth of *A. africana* Roots

Regenerated axillary shoots (20–30 mm) from stem nodal segments were excised and transferred to 100 ml full strength MS supplemented with three different auxins—naphthaleneacetic acid (NAA), indole-3-acetic acid (IAA), and indole-3-butyric acid (IBA)—at concentrations of 0.1, 0.25, 0.5, 0.75, and 1.0 mg/l. Two axillary shoots per culture vessel (125 × 110 mm; Figure 1E) with 20 replicates were used for each treatment. The rooting rate ($\frac{N\,u\,m\,b\,e\,r\,o\,f\,s\,h\,o\,o\,t\,s\,t\,h\,a\,t\,f\,o\,r\,m\,e\,d\,r\,o\,o\,t\,s\,i\,n\,r\,o\,o\,t\,i\,n\,g\,m\,e\,d\,i\,u\,m}{T\,o\,t\,a\,l\,n\,u\,m\,b\,e\,r\,o\,f\,p\,l\,a\,n\,t\,s\,h\,o\,o\,t\,s\,i\,n\,o\,c\,u\,l\,a\,t\,e\,d\,i\,n\,t\,h\,e\,r\,o\,o\,t\,i\,n\,g\,m\,e\,d\,i\,u\,m}$ × 100), number of roots, and root lengths were determined after 6 weeks of culture.

### Acclimatization of Regenerated Plants

Traces of media on the roots of *A. africana* regenerated plantlets were removed (Figure 1F) by thorough rinsing under flowing tap water, and the plantlets were transferred to plastic pots (13 × 11 cm) containing a mixture of sterile horticulture soil and perlite (2:1 ratio). The plantlets in the plastic pots were enclosed in transparent polythene bags to ensure sufficient humidity (Figure 1G) and were maintained in a greenhouse. After 12 days, the polythene bags were gradually opened as the plants acclimatized. The plants were watered once weekly using tap water before removal of the transparent polythene bags and then twice weekly after removal of the polythene bags. After acclimatization for 7 weeks (Figure 1H), the survival rate of the plants was determined.

### Measurement of Chlorophyll Content

**FIGURE 2:**
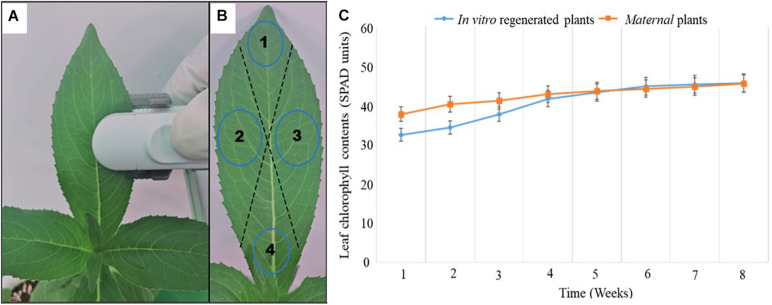
Chlorophyll content measurements of *A africana in vitro* regenerated and maternal plant leaves. **(A)** Chlorophyll content of leaf being measured using SPAD instrument. **(B)** Different points/regions on a leaf considered for the average chlorophyll content of each leaf. **(C)** Chlorophyll contents of *in vitro* generated *A africana* plants compared to that of the maternal plants over a period of 8 weeks.

Leaf chlorophyll content of *in vitro* regenerated *A. africana* plants after 4 weeks of acclimatization was compared with that of the mother plants using a calibrated Soil Plant Analysis Development chlorophyll meter (SPAD-502 Plus, Konica Minolta, Inc., Japan). Eight leaves (one pair from the apical region, two pairs from mid-stem, and one pair from lower stem regions) on each plant were randomly selected, and SPAD chlorophyll content readings were taken from four different regions of each leaf to obtain the average value for that leaf (Figures 2A,B). The average value of SPAD readings for all leaves (8) was considered the average leaf chlorophyll content of that plant. Chlorophyll contents of the same plants (20 plants: 10 *in vitro* regenerated and 10 mother plants) were measured weekly over a period of 8 weeks (Figure 2C).

### Chlorophyll Fluorescence Measurement

**FIGURE 3:**
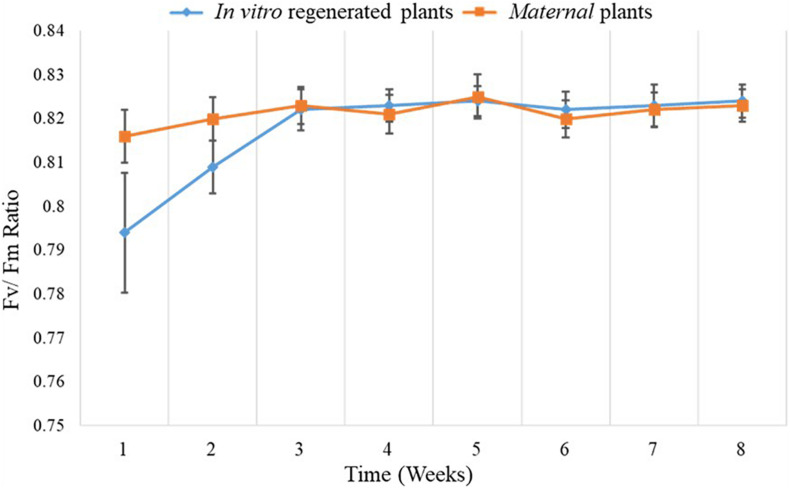
Comparison of mean Fv/Fm ratio of *in vitro* regenerated and maternal *A. africana* plants over a period of 8 weeks.

Chlorophyll fluorescence measurements were performed using FluorPen FP110 (Drásov 470, 664 24 Drásov, Czechia) after adapting the leaves to darkness with leaf clip gaskets for approximately 1 h. Ten (10) leaves were randomly selected from each of the ten replicates of *in vitro* regenerated and mother plants, and their chlorophyll florescence was measured. Chlorophyll fluorescence data were collected weekly over a period of 8 weeks (Figure 3). A saturating flash beyond 4,000 μmol m^–2^ s^–1^ was used to obtain Fv/Fm values based on the FluorPen FP110 OJIP protocol. In the dark-adapted state, minimal intensity of chlorophyll fluorescence (Fo), maximal intensity of chlorophyll fluorescence (Fm), and variable chlorophyll fluorescence (Fv) were measured when all reaction centers for photosystem II (PSII) were open, during application of saturation light pulse, and when there were minimal non-photochemical processes, respectively.

### Fourier Transform Near-Infrared (FT-NIR) Analysis

**FIGURE 4:**
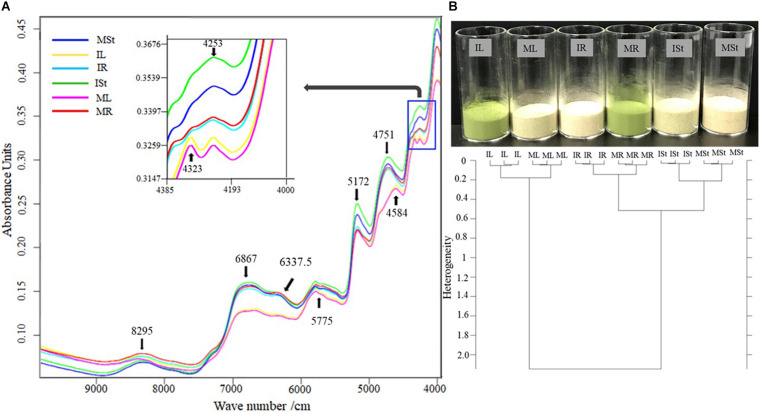
**(A)** Comparison of FT-NIR spectral lines of samples from different parts of *in vitro* regenerated and maternal *A. africana* plants. **(B)** Clustering Dendrogram for the different samples of *in vitro* regenerated and maternal *A. africana* plants analyzed from FT-NIR. *In vitro* regenerated *A. africana* plant samples analyzed: IL-leaf, ISt-stem, and IR-root. Maternal *A. africana* samples analyzed: ML-leaf, MSt-stem, and MR-root.

Different *A. africana* plant parts from the *in vitro* regenerated [IL (leaf), ISt (stem), and IR (root)] and mother plants [ML (leaf), MSt (stem), and MR (root)] were collected, oven-dried (at 60°C for 48 h), and then pulverized to a fine powder using 250G Pulverizing Machine at 25,000 rpm (Model RT-N04-2V, Taiwan). The analysis was performed using a TANGO FT-NIR spectrometer (Bruker Optics, Billerica, MA, United States). Calibration of the spectrometer was performed with a Light Trap (Type 1002961, ECL 00 and Gold standard; Type 1024957, ECL:01), and then 2 g of each powdered sample in a vial (20 mm in diameter) was analyzed. The absorbance spectra were obtained at 12,487–3,948 cm^–1^ wavenumbers, to determine the different classes of compounds in the samples based on their functional groups (Figure 4A). Dendrograms for the samples were constructed on the basis of Ward’s algorithm clustering upon characteristic preprocessing of data (first derivative) and vector normalization and standardizing the Euclidean distance at the 9,981–4,014 cm^–1^ frequency range (Figure 4B). The OPUS TANGO-R software was used for the Ward algorithm. Homogeneous categories were maximally sorted using the minimum variance method analysis of clusters.

### Anatomical Comparison of *in vitro* Regenerated and Mother Plants

**FIGURE 5:**
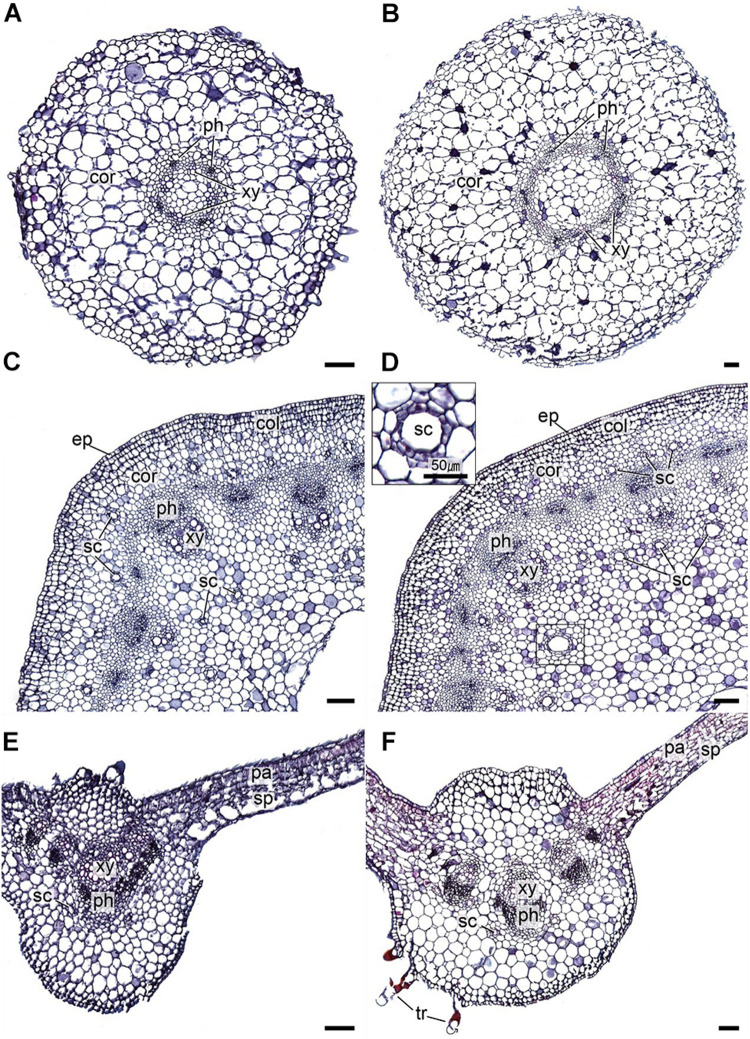
Transverse sections of *A. africana in vitro* regenerated and maternal plant tissues. **(A)** *In vitro* plant root. **(B)** Maternal plant root. **(C)** *In vitro* plant stem. **(D)** Maternal plant stem. **(E)** *In vitro* plant leaf. **(F)** Maternal plant leaf. cor, cortex; xy, xylem; ph, phloem; ep, epidermis; col, collenchyma; sc, secretory duct; pa, palisade parenchyma; sp, sponge parenchyma; tr, trichome. Scale bars = 100 pm.

Detailed anatomical comparison of leaf, stem, and root structures of the *in vitro* regenerated and maternal *A. africana* plants was performed by microscopic analysis of the tissues. The *A. africana* plant tissues were dehydrated successively for 1 h in 50, 70, 80, 90, 95, 98, and 100% ethanol at 25 ± 2°C. The dehydrated tissues were then cleared for 30 min each at 25 ± 2°C in 75% ethanol, 25% xylene; 50% ethanol, 50% xylene; 25% ethanol, 75% xylene; and then twice in 100% xylene. This was followed by paraffin embedding at 60°C for 1 h each in 2/3 xylene:1/3 paraffin, 1/3 xylene:2/3 paraffin, paraffin, and paraffin before embedding overnight in paraffin. The paraffin-embedded tissues were sectioned into 12-μm slices using a sliding microtome (SM2010R, Leica Biosystems Nussloch GmbH, Heidelberger Str. 17-19 D-69226 Nussloch, Germany). The tissues were deparaffinized and then rehydrated for 5 min successively in xylene (twice), 50% ethanol, 50% xylene, 100% ethanol (twice), 95% ethanol, 70% ethanol, and 50% ethanol. The dehydrated *A. africana* tissues were stained as follows: 1% safranin (1 h), rinsed in water; 50% ethanol (3 min), 70% ethanol (3 min), and 95% ethanol (3 min); fast-green (3 min); twice in 100% ethanol (1 min each); carbol-xylene (30 min); three times in xylene for 5, 15, and 15 min. Images of the stained tissues (Figure 5) were then captured using a light microscope (Olympus BX-53, Tokyo, Japan) and a digital camera (Olympus DP21, Olympus, Tokyo, Japan) after mounting with Balsam medium.

### Statistical Analysis

All experimental data were subjected to one- or two-way analysis of variance (ANOVA) with Tukey’s *post hoc* tests using Prism (Graph Pad software, v 5. 03). All means compared were considered significantly different at *p* ≤ 0.05.

## Results

### Effect of Media on the Growth of *A. africana* Shoots

**FIGURE 6:**
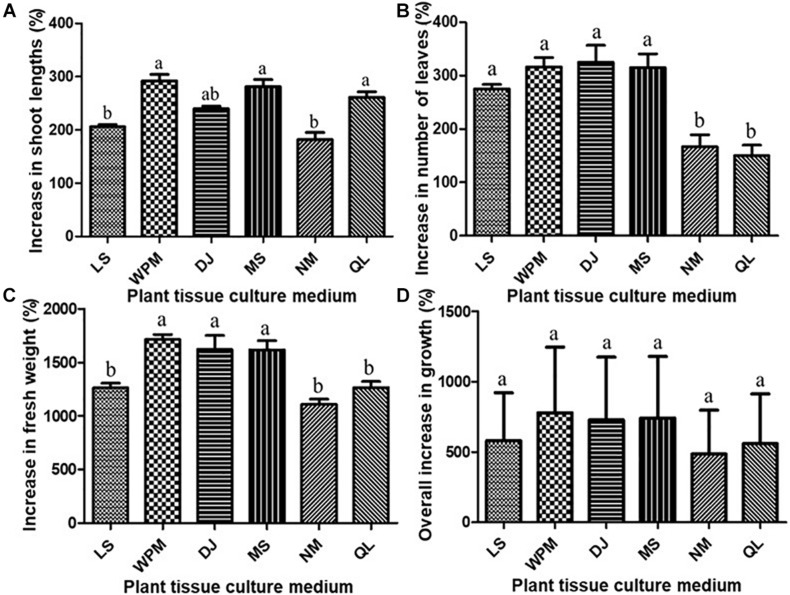
Plant tissue culture media effects on shoot growth of *A. africana* plants from shoot tip explants. **(A)** Effect on shoot lengths. **(B)** Effect on leaf numbers. **(C)** Effect on fresh weights. **(D)** Effect on overall growth indices. Same letter are not significantly different by Tukey’ s test and *p* = 0.05.

All media investigated favored the shoot regeneration and growth of *A. africana* from its shoot tips based on the parameters measured after 6 weeks of growth (Figure 6). The highest percentage increase in the shoot length of *A. africana* plants was 292.1 ± 11.93% in WPM; however, it did not differ significantly from the percentage increase in MS (280.7 ± 13.90), QL (261.0 ± 10.38%), or DJ (239.4 ± 5.14%) (Figure 6A). The lowest percentage increase in the shoot length of *A. africana* was 182.0 ± 13.16% in NM medium (Figure 6A). The shoots of *A. africana* in DJ medium showed the highest increase in number of leaves at 325.0 ± 31.84%; however, this did not significantly differ from the increase in MS (315.0 ± 36.66%), WPM (316.7 ± 17.21%), or LS medium (275.0 ± 8.33%) (Figure 6B). The leaves of *A. africana* plants in MS medium appeared dark green and healthier. The lowest increase in the number of leaves was 150.0 ± 19.72% recorded in the QL medium, and it did not significantly differ from the increase in NM medium (166.7 ± 22.36%) (Figure 6B). All media investigated produced over a 1,000% increase in the fresh weight of *A. africana* shoot cultures (Figure 6C). The percentage increase in fresh weights of *A. africana* shoot cultures in WPM, DJ, and MS was significantly higher (*p* < 0.05) than that in the LS, NM, and QL media (Figure 6C). Overall, there were no significant differences in the growth indices of *A. africana* in the different media tested (Figure 6D). Compared to WPM, which had the highest overall growth index, MS was chosen as the ideal medium for growth of *A. africana* and for use in subsequent experiments because the plants were generally healthier with broader and darker green leaves.

### Effects of Cytokinins on *A. africana* Shoot Formation and Proliferation

**FIGURE 7:**
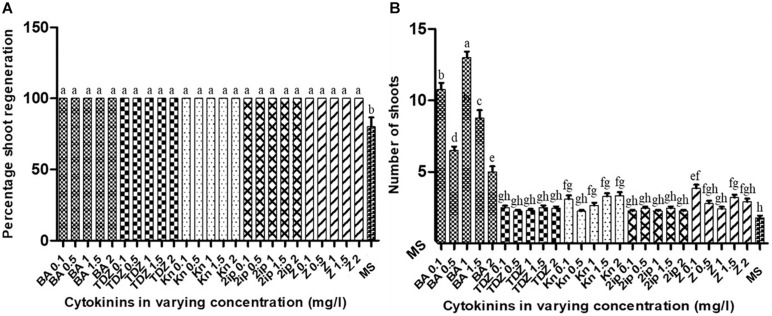
Effects of different cytokinins on *in vitro* shoot regeneration of *A. africana* from nodal explants. **(A)** Effects on percentage regeneration. **(B)** Effects on of shoot numbers formed per nodal explants. Same letters are not significantly different by Tukey’s test and *p* = 0.05.

The nodal segments of *A. africana* in MS medium supplemented with all concentrations of cytokinins (BA, TDZ, Kn, 2iP, and zeatin) produced 100% shoot formation; the percentage of shoot formation significantly differed (*p* < 0.05) from that in MS without any cytokinin (control) (Figure 7A). Shoot formation and multiplication from *A. africana* nodal segments were highly favored in MS medium supplemented with BA in comparison to all other cytokinins investigated (Figure 7B). Media supplemented with all BA concentrations, except at 2.0 mg/l, had significantly higher (*p* < 0.05) number of *A. africana* shoots than those supplemented with TDZ, Kn, 2iP, or zeatin at all concentrations tested (Figure 7B). MS medium with 1.0 mg/l BA produced the highest mean number of *A. africana* shoots (13.0 ± 0.424), which was significantly higher (*p* < 0.05) than the mean number of shoots produced with all other concentrations of BA and all other cytokinins (Figure 7B). MS medium supplemented with 0.5 mg/l BA produced the second highest number (10.75 ± 0.486) of shoots from nodal segments, followed by 1.5 mg/l BA (6.5 ± 0.267). Proliferation was the lowest in BA at the highest concentration (2.0 mg/l) compared to all other BA concentrations (Figure 7B). Shoot proliferation from *A. africana* nodal segments in MS medium supplemented with TDZ, Kn, 2iP, or zeatin at any concentration did not significantly differ from that in the control.

### Effects of Auxins on Rooting of *A. africana* Shoot

**FIGURE 8:**
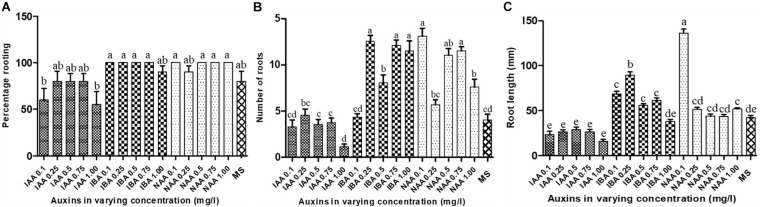
Effects of auxins on *in vitro* rooting of regenerated *A. africana* shoots. **(A)** Effects on percentage rooting. **(B)** Effects on number of roots formed per shoot. **(C)** Effects on root lengths. Same letters are not significantly different by Tukey’s test and *p* = 0.05.

All regenerated *A. africana* shoots transferred to rooting media with or without auxins subsequently rooted. However, there were significant differences in rooting percentage, root number, and root length (Figure 8). Maximum rooting percentage (100%) of regenerated shoots occurred in media supplemented with IBA (0.1, 0.25, 0.50, and 0.75 mg/l) and NAA (0.1, 0.50, 0.75, and 1.0 mg/l); however, these were not significantly higher than the rooting percentages in any other treatments, including the control (MS), except in 0.1 mg/l IAA and 1.0 mg/l IAA (Figure 8A). The rooting percentage was the lowest in media supplemented with the IAA auxin among all the auxins tested (Figure 8A). The highest number of roots was 13.10 ± 0.873 obtained in MS medium with 0.1 mg/l NAA, followed by 12.55 ± 0.634 in 0.25 mg/l IBA, and then 12.10 ± 0.602 in 0.75 mg/l IBA (Figure 8B). The root number in media supplemented with 0.1, 0.5, and 0.7 mg/l NAA and 0.25, 0.75, and 1.0 mg/l IBA was significantly higher (*p* < 0.05) than that in all other treatments (Figure 8B). The lowest mean number of roots (1.15 ± 0.264) was recorded in MS medium supplemented with 1.0 mg/l IAA (Figure 8B). The longest roots were recorded with 0.1 mg/l NAA, with a mean of 136.35 ± 4.316 mm, which was significantly higher (*p* < 0.05) than the mean root lengths obtained with all other treatments (Figure 8C).

### Acclimatization of Regenerated *A. africana* Plants

After 7 weeks of acclimatization, *in vitro* regenerated *A. africana* plantlets had a survival rate of 95.7%. The acclimatized plants grew healthily. Compared to the maternal *A. africana* plants, the acclimatized *in vitro* regenerated plants showed no evident variation in morphology or growth characteristics.

### Chlorophyll Content Assessment

During the first week, chlorophyll contents of *in vitro* regenerated plants (32.5 ± 1.734) were much lower than those of the maternal plants (37.8 ± 1.740) (Figure 2C); however, by the fourth week, the SPAD chlorophyll content of *in vitro* regenerated plants had risen slightly faster than that of the maternal plants and reached almost the same level (Figure 2C). From the fifth to the eighth week, there was only a slight rise in chlorophyll contents of both *in vitro* regenerated plants (from 43.33 ± 1.328 to 45.75 ± 1.343) and mother plants (from 43.67 ± 1.489 to 45.58 ± 1.384) (Figure 2C). The chlorophyll contents of *in vitro* regenerated and mother plants were comparable over the last 4 weeks of measurement (Figure 2C).

### Chlorophyll Fluorescence Assessment

To evaluate the photosynthetic performance of *in vitro* regenerated and maternal plants, the dark-adapted Fv/Fm values (indicating the maximum or intrinsic potential quantum efficiency of PSII) were measured. The Fv/Fm values of the *in vitro* regenerated plants ranged from 0.821 ± 0.0019 to 0.830 ± 0.0015, while those of the maternal plants ranged from 0.822 ± 0.0022 to 0.834 ± 0.0017 during the 8 weeks of measurement (Figure 3). The Fv/Fm values of the *in vitro* regenerated and maternal plants were within the same range, with only marginal variation, particularly from the third to eighth week of measurement (Figure 3).

### FT-NIR Assessment

Generally, the spectra for stem and root samples (both *in vitro* regenerated and maternal plants) were similar, but slightly different from the spectra of leaves between 5,000 and 4,000 cm^–1^ wavenumbers (Figure 4A). For all the stem and root samples, a peak at 4,751 cm^–1^ was observed, which did not appear in the leaf samples (Figure 4A). Only the root samples (both *in vitro* regenerated and maternal plants) showed peaks at 4,584 and 4,323 cm^–1^ (Figure 4A). For both *in vitro* regenerated and maternal plant samples, eight prominent peaks for leaves (IL and ML) and seven prominent peaks for roots (IR and MR) and stems (ISt and MSt) between 9,000 and 4,000 cm^–1^ wavenumbers were observed in the FT-NIR spectra (Figure 4A). Clustering of samples based on Ward’s algorithm showed a heterogeneity value of 0.52 for the stems (ISt and MSt) and roots (IR and MR) (Figure 4B). The leaves (IL and ML) had a small heterogeneity (0.17) from each other, but showed a high dissimilarity with the stems and roots, with a heterogeneity value of 2.14 (Figure 4B). The *in vitro* regenerated and mother plant parts had very close similarity with each other, with heterogeneity values of 0.17, 0.21, and 0.14 for leaves, stems, and roots, respectively (Figure 4B).

### Anatomical Comparison of *in vitro* Regenerated *A. africana* and Mother Plants

Detailed anatomical examination and comparison of cross sections of *in vitro* regenerated and maternal root, stem, and leaf tissues were carried out. Root cross sections of the *in vitro* regenerated and maternal plants revealed that the epidermis in both was 1–2 cells thick and consisted of rectangular and oval cells, but the thickness of epidermal layers was 7.42–37.0 μm (*in vitro* regenerated) and 16.67–62.31 μm (maternal plants) (Figures 5A,B). Parenchymatic cortex in both comprised 8–12 layers of oval cells of different sizes, but cortex widths in the *in vitro* regenerated and maternal plant roots ranged between 360.13–432.0 μm and 666.64–916.32 μm, respectively (Figures 5A,B). The endodermis of both was made up of 1–2 layers of oval and rectangular cells and the vascular bundles were of the radial type with distinct phloem and xylem tissues in both (Figures 5A,B). Unlike in the maternal plant root, a few root hairs, 33–67 μm long, projected from the root epidermal layer of *in vitro* regenerated plants (Figures 5A,B).

Through the stem cross sections of both *A. africana in vitro* regenerated and maternal plants, the following observations were made: the epidermis of both consisted of 1–2 layers of rectangular and oval cells, with thickness ranging from 10.71–39.74 μm and 15.64–54.31 μm in *in vitro* regenerated and maternal plant stems, respectively (Figures 5C,D). The cortex in both consisted of 7–10 layers of differently sized oval parenchymatic cells with thickness of 160.71–235.7 μm (regenerated) and 159.09–254.5 μm (maternal) (Figures 5C,D). In both, the sclerenchymatic caps just above the phloem were made up of 3–6 cell layers, secretory ducts were abundantly present in the cortex and pith of both regenerated and maternal plant tissues (diameter of secretory ducts was 7.143–25.0 μm (regenerated) and 9.09–81.82 μm (maternal) (Figures 5C,D), and vascular bundles were medullary with prominent phloem and xylem tissues. Xylem and phloem cells covered almost equal areas, together forming open-type collateral vascular systems in both (Figures 5C,D), and both had very wide piths consisting of differently sized polygonal and oval parechymatous cells (Figures 5C,D).

Anatomical features of the leaf lamina base of the *in vitro* regenerated and maternal plants at lengths of about 750 μm from the midrib revealed that the adaxial and abaxial epidermises in both were uniseriate consisting of rectangular cells; however, abaxial epidermal cells in the *in vitro* regenerated plant tissue were poorly differentiated (Figures 5E,F). The mesophyll palisade consisted of a single layer of tightly packed elongated parenchyma palisade cells in the maternal tissue, while in the *in vitro* regenerated tissues, the palisade cells were unorganized and poorly developed (Figures 5E,F). The spongy mesophyll consisted of irregularly shaped and arranged parenchyma cells with narrow intercellular air spaces in the maternal tissue, while in *in vitro* regenerated plants, the mesophyll tissue consisted of poorly differentiated parenchyma cells with very wide air spaces (Figures 5E,F). The detailed anatomy of the midrib cross-section in both showed that the midribs were elevated in the middle, being more or less acutely angled with leaf blades (Figures 5E,F), and the epidermises were uniseriate consisting of largely irregularly shaped and differently sized epidermal cells. The cells in the epidermis of *in vitro* regenerated plant tissues were poorly differentiated, while those of the maternal tissue were well-differentiated (Figures 5E,F); additionally, the parenchymatous cells were of various shapes and sizes, with most being spherical or polygonal (Figures 5E,F). Five (5) vascular bundles, consisting of phloem and xylem tissues, were present in each cross section, with the largest at the midrib center (Figures 5E,F). Secretory ducts were present in both tissues (Figures 5E,F) and trichomes were clearly more visible through the mid-rib cross section of the maternal *A. africana* plants than in the *in vitro* regenerated plants (Figures 5E,F).

## Discussion

Plant tissue culture media, as the source of mineral nutrients and water to the plants, plays a critical role in the *in vitro* propagation of plants (Komakech et al., 2020). Plant media are formulated differently, and the variation in their composition has different effects on plant growth (Kazemi and Mohorko, 2017; Komakech et al., 2020). The main differences among culture/growth media are the macronutrients (including nitrate and ammonium ions) and total ion concentrations. For instance, MS has very high nitrate (39.4 mM) and ammonium (20.6 mM) but low sulfate (1.7 mM) ion contents, QL has low ammonium ion content (5.0 mM), and WPM has low ammonium (5.0 mM) and nitrate (9.7 mM) but high sulfate (7.5 mM) ion contents (Bell et al., 2009; Carlín et al., 2015). Although there were significant differences in particular growth parameters, the overall growth index of *A. africana* in the tested media did not differ significantly because all the media had sufficient nutrients for the growth of plants.

In this study, MS was selected as the optimum medium for growth of *A. africana*, although it had the second highest overall increase in growth index based on all the parameters considered. This was because compared to that in WPM, in which *A. africana* plant growth was the highest, though not significantly higher than that in MS, the *A. africana* plants in MS had darker leaves and looked healthier. Leaf color is an indicator of the nutritional and health status (Gupta et al., 2013). The dark green leaves and healthy appearance of *A. africana* in MS could be attributed to the high amount of nitrogen in the medium (Bonnecarrère et al., 2009). Nitrogen is important for chlorophyll formation, photosynthetic efficiency, and stomatal conductance and contributes up to 41% of plant growth (Ivonyi et al., 1997; Ohshiro et al., 2016). Furthermore, a number of plants in the same family Asteraceae, including *Aspilia mossambicensis* (Norton et al., 1993), *Blumea mollis* (Tamilarasi and Thirugnanasampandan, 2014), *Eupatorium triplinerve* (Janarthanam et al., 2011), and *Achillea filipendulina* (Evenor and Reuveni, 2004), have also been successfully regenerated in MS medium. However, some studies have indicated that the growth of other plants, particularly the woody members of the Asteraceae family such as *Achyrocline satureioides* (Guariniello et al., 2018) and *Achyrocline flaccida* (Bonnecarrère et al., 2009), is better in WPM.

Cytokinins generally facilitate shoot multiplication and elongation in plants (Kim et al., 2021). Previous studies on *in vitro* regeneration from nodal explants of a number of species in the Asteraceae family indicated maximum shoot proliferation when plant tissue culture media were supplemented with only cytokinin(s) without auxins for instance, *Stevia rebaudiana* (Ahmed et al., 2007; Rafiq et al., 2007; Thiyagarajan and Venkatachalam, 2012), *Artemisia vulgaris* (Sujatha and Kumari, 2008), *Spilanthes acmella* (Singh and Chaturvedi, 2010; Kurian and Thomas, 2015), *Eclipta alba* (Borthakur et al., 2000), and *Spilanthes calva* (Razaq et al., 2013). In the present study, BA was the most effective for shoot proliferation from nodal segments of *A. africana* among all the cytokinins tested. Similar observations were made for other plants including *Ipomoea batatas* (Dewir et al., 2020), *Crataeva nurvala* (Kher and Nataraj, 2020), and *Psiadia arguta* (de Souza et al., 2007). The ability of BA to initiate cell division and development of lateral buds makes it superior to other cytokinins, as it plays a key role in breaking bud dormancy (Revathi et al., 2019; Dewir et al., 2020; Kher and Nataraj, 2020; Rajput et al., 2020). de Souza et al. (2007) also observed that lower concentrations of BA (0.25 and 0.5 mg/l) were superior in shoot multiplication of *P. arguta*, just as we observed in *A. africana*. At high concentrations of BA, vitrification occurs, which causes hyperhydricity, interfering with normal shoot multiplication and growth (Komakech et al., 2020). This could have resulted in a reduced tendency for shoot proliferation at high concentrations of BA (above 2.0 mg/l). Wang et al. (2018) further indicated that an increase in cytokinin concentration above the optimal level negatively affects shoot regeneration. However, a study by Kurian and Thomas (2015) revealed that, contrary to our observation, a high concentration (2.0 mg/l) of BA was the most effective for *Spilanthes acmella* shoot proliferation from its nodal explants. This implies that different plant species have varying tolerance to the cytotoxic effects of exogenous cytokinins. In our study, among all the concentrations of BA studied, the highest proliferation rate was 1.0 mg/l, with an average of 13 shoots developed from a nodal segment over a period of 6 weeks (Figure 7). In the shoot proliferation study on *Stevia rebaudiana*, another plant in the Asteraceae family, 1.0 mg/l BA was also observed to produce the highest shoot number of 15.69 per explant (Thiyagarajan and Venkatachalam, 2012). In another study where different hormones, either singly or in combination, were tested for shoot proliferation of *Spilanthes calva*, 1.15 mg/l BA was selected as the optimum proliferation medium, producing up to 4.17 shoots per explant (Pandey et al., 2014). Pereira et al. (2013) also showed that a low concentration of 1.11 mg/l BA resulted in optimum shoot proliferation (8.7%) from *Lychnophora ericoides* nodal segments. Similar to our findings, 0.5 mg/l BA had overall highest proliferation rate of *Anthemis xylopoda*, producing 6.7 shoots per explant compared to various concentrations of Kn and TDZ (Erdaǧ and Emek, 2009). Unlike in our study, a combination of cytokinins with auxins proved effective in shoot proliferation from nodal segments in a number of plants of the Asteraceae family, including *Eclipta alba* (1.0 mg/l Kn and 3.0 mg/l BA) (Singh et al., 2012), *Achillea occulta* (0.5 mg/l IBA and 1.1 mg/l BA) (Constantinidis and Kalpoutzakis, 2009), and *S. rebaudiana* (0.5 mg/l Kn and 1.5 mg/l BA) (Ahmed et al., 2007). Overall, as discussed above, plant species respond differently to various cytokinins, although in general, members of the Asteraceae family seem to respond well to BA as opposed to other cytokinins.

Auxins play an important role in root initiation and growth (Komakech et al., 2020). Rooting of *A. africana in vitro* regenerated shoots was greatly influenced by auxin type and concentration. A number of studies have also reported varying rooting responses to different auxins from other regenerated plant shoots, even within the same genus (Feyissa et al., 2005; Schoene and Yeager, 2005). *Aspilia africana* regenerated shoots responded well to IBA and NAA auxins, with rooting percentages of 100% in all but one of the five tested concentrations (0.1–1.0 mg/l) for both auxins. A significant (*p* < 0.05) rooting response was observed in medium containing 0.1 mg/l NAA, with 13.10 ± 0.873 roots at an average length of 136.35 ± 4.316 mm (Figure 8). The auxin NAA was also shown to be best for rooting, in a number of other studies, in various plants, including *S. rebaudiana* (Rafiq et al., 2007), *Dendranthema grandiflorum* (Khandakar et al., 2014), and *L. pinaster* (de Souza et al., 2007). Similar to our study, Ahmed et al. (2007) reported that 0.1 mg/l NAA was the best for rooting of *S. rebaudiana*, with the highest rooting percentage of 97.66%. Furthermore, a study on *D. grandiflorum* also reported that the *in vitro* regenerated shoots developed roots well in 0.1 mg/l NAA (Khandakar et al., 2014). *Aspilia africana* root numbers and lengths were reduced at high concentrations of NAA, possibly due to auxin inhibition effects, as has been reported for *Arabidopsis* (Ivanchenko et al., 2010). In our study, the second-best rooting of *A. africana* plants was in 0.75 mg/ml IBA. Similarly, other studies on related plant species also reported that IBA was ideal for rooting of *in vitro* regenerated shoots, including those of *Elephantopus scaber* (Abraham and Thomas, 2016), *A. xylopoda* (Erdaǧ and Emek, 2009), *Echinacea purpurea* (Koroch et al., 2002), and *Centaurea cineraria* (Valletta et al., 2016). Contrary to our findings, a few related plant species, such as *S. rebaudiana* (Ahmed et al., 2007) and *Bellis perennis* (Karakas and Turker, 2013), responded best to rooting in IAA. The very low rooting activity of IAA in our study could partly be attributed to its decomposition resulting from autoclaving since it is relatively less stable compared to IBA and NAA (Nissen and Sutter, 1990).

Acclimatization of rooted *in vitro* regenerated plants is an important component of their subsequent field survival, as these plants tend to possess abnormal anatomy, physiology, and morphology and, thus, require time to acclimatize (Komakech et al., 2020). We observed that the *in vitro* regenerated *A. africana* had a very high survival rate of 95.7% during the acclimatization period. Similar high survival rates of other plant species in the same family have also been recorded, including 100% survival for *E. scaber* (Abraham and Thomas, 2016), 90–100% for *E. alba* (Singh et al., 2012), and 98.4% *for S. rebaudiana* (Hwang, 2006).

Chlorophyll is an important photosynthetic pigment that largely determines the photosynthetic capacity, health, and growth of a plant (Li et al., 2018). It harvests sunlight at various wavelengths to excite electrons, which drives chemical energy and nicotinamide adenine dinucleotide phosphate formation (Croft et al., 2017). The use of an SPAD meter for chlorophyll measurement is a non-destructive, rapid, and accurate method (Ling et al., 2011). A number of studies have confirmed that leaf chlorophyll content values measured using the SPAD meter are positively correlated with other destructive methods of chlorophyll content measurement (Rodriguez and Miller, 2000; Coste et al., 2010). In the first week of measurement, the leaf chlorophyll content of *in vitro* regenerated *A. africana* plants was much lower than that of the mother plants. The low chlorophyll content was probably due to the decreased photochemical activity of *A. africana in vitro* leaves in the first weeks of acclimatization, as was also observed in *Tecoma stans* L. (Hussain et al., 2020). In addition, the *in vitro* regenerated *A. africana* plants had younger leaves, and the plants were just adapting to the *in vivo* environment. Mature leaves have more stable and higher chlorophyll content than the young leaves, as has been demonstrated in *Prunus africana* (Komakech et al., 2020), *Psidium guajava* L., and *Mangifera indica* L. (Kamble et al., 2015). As the *in vitro* regenerated *A. africana* plants obtained minerals from soil, adapted, and their leaves matured, photochemical activity increased and chlorophyll content increased rapidly. By the fifth week, chlorophyll levels were almost at the same level as in the maternal plants. Soil provides essential elements, such as nitrogen and phosphorus, to plants for the synthesis of chlorophyll (Li et al., 2018). In the last 4 weeks of chlorophyll measurement, leaf chlorophyll contents in both *in vitro* regenerated and maternal *A. africana* plants were relatively stable and almost the same, indicating similarity in their photosynthetic rates, as was previously observed in *P. africana* (Komakech et al., 2020).

The dark-adapted Fv/Fm values are used as a reliable indicator of photosynthetic potential in plants (Murchie and Lawson, 2013). Chlorophyll fluorescence assessment in PSII has been used in a number of studies to determine the photosynthetic rate in various plants, including *Triticum turgidum* L. (Moustaka et al., 2018), *P. africana* (Komakech et al., 2020), and *Pseudotsuga menziesii* (Perks et al., 2001). The lower Fv/Fm values in the first 2 weeks of measurement for *in vitro* regenerated plants could be attributed to poorly differentiated and underdeveloped photosynthetic tissues of *in vitro* plantlets (Shekhawat and Manokari, 2018). When *in vitro* regenerated plants are exposed long enough to the external environment, the leaf mesophyll differentiates and tissues become more developed, thus fully adapting the plants for photosynthesis (Abd El-Zaher, 2008; Batagin-Piotto et al., 2012; Shekhawat and Manokari, 2018). The Fv/Fm values of the *in vitro* regenerated *A. africana* plants from the third until the last week of measurements ranged from 0.821 ± 0.0047 to 0.824 ± 0.0037 and those of the maternal plants ranged from 0.823 ± 0.0042 to 0.823 ± 0.0037, indicating that they had similar photosynthetic rates. The Fv/Fm values recorded were similar to those recorded for other plants, including 41 different wheat cultivars (Sharma et al., 2015), *Solidago canadensis* (Immel et al., 2012) and *Inula montana* (Roux et al., 2017). The Fv/Fm values normally range from 0.75 and 0.85 for plants that are not under any stress (Roux et al., 2017). This indicates that both the *in vitro* regenerated and maternal *A. africana* plants grew under favorable conditions with no stress.

FT-NIR spectroscopy is a non-destructive chemical assessment technology that has been widely used for the identification and characterization of chemical compounds in a wide range of samples (Komakech et al., 2020; Wang et al., 2020). The FT-NIR spectra provide information about the major chemical bonds from which the chemical composition of the samples can be deduced (Wang et al., 2020). In this study, the broad band at 8,295 cm^–1^ wavenumber was due to the second overtone of C-H stretching vibrations. Stretching vibrations are linked to CH_2_ and CH_3_ groups (Wang et al., 2020). The absorption peaks from 7,000 to 6,300 are due to the overtones of O-H stretching. The overtone of O-H stretching modes originates from phenolic groups, carboxyl O-H groups, starch, and water (Kirchler et al., 2017; Komakech et al., 2020). The absorbance at 5,775 cm^–1^ originated from the C–H stretching modes of aliphatic chains and aromatic rings. The sharp peak at 5,172 cm^–1^ corresponded to O-H stretching originating from water. Peaks within 5,000–4,500 cm^–1^ originated from a combination of N-H, C-H stretching, and O-H stretch mode associated with proteins. The absorbance peak at 4,336 cm^–1^ was possibly due to a combination of C-H stretching and ring deformation, whereas the peak at 4,253 cm^–1^ might be mainly due to aliphatic and aromatic C-H stretching. The similarity in the spectra of *in vitro* regenerated and maternal *A. africana* samples signifies homogeneity in terms of chemical composition of *in vitro* regenerated and mother *A. africana* plants. In addition, Ward’s algorithm was used to cluster leaf, stem, and root samples from *in vitro* regenerated and maternal *A. africana*. Ward’s algorithm clustering is widely used to characterize a wide range of samples, including plant samples (Komakech et al., 2020). The roots (IR and MR) and stems (ISt and MSt) of *A. africana* had a higher degree of homogeneity to each other than to the leaves (IL and ML) and formed the first cluster at 0.52% (Figure 4B). The *in vitro* regenerated and maternal *A. africana* plant parts showed very close similarity with roots having the lowest heterogeneity at 0.14, followed by leaves (0.17) and stems (0.21) (Figure 4B). The high degree of homogeneity in the samples could be attributed to the similarity in chemical composition, as depicted in the FT-NIR spectra of the samples (Figure 4A). This resulted in a smaller distance separation of the samples, as displayed in the dendrogram (Figure 4B). Different plant parts may comprise different major compounds, which contribute to the heterogeneity in these parts. Similarly, Komakech et al. (2020) in their FT-NIR assessment of *P. africana* observed similar trends, with roots of *in vitro* regenerated and mother *P. africana* plants being the most closely homogeneous at 0.21; however, unlike in our case, the roots were followed by stems (0.28) and then leaves (0.67). The small degree of heterogeneity of similar parts could be attributed to the age difference between the *in vitro* regenerated and maternal *A. africana* plants. Plant chemical composition is influenced by age (Achakzai et al., 2009; Rencoret et al., 2011). Similar studies also confirmed, through Ward’s algorithm clustering, that heterogeneity exists when samples from plants of different ages are compared (Srivastava et al., 2018; Komakech et al., 2020).

A detailed anatomical comparison between the *in vitro* regenerated and maternal *A. africana* plants showed very close similarity in root and stem tissues, with both having similarly well-differentiated and fully developed structures that only varied in thickness and size. The variation in sizes of the tissue structural components could be due to the differences in size and age of the *in vitro* regenerated and maternal *A. africana* plant parts compared. Similar observations were made by Shekhawat and Manokari (2018) who noted that *in vitro* regenerated plants possessed smaller leaves, thinner stems, and roots. However, during acclimatization, these plant parts grew thicker and larger (Shekhawat and Manokari, 2018). The presence of well-developed and fully differentiated plant tissues may be related to their functionality (Kim et al., 2021). Differentiated tissues, such as vascular bundles in roots and stems, indicate that these organs were fully developed and functional. This is also supported by the fact that the *in vitro* regenerated plants had a high survival rate during acclimatization. Paunescu (2008) noted that the *in vitro* regenerated plants whose tissues were underdeveloped and not fully differentiated had very low survival chances during acclimatization. Similarly, i*n vitro* regenerated *Coccinia indica* plant stem and root tissues fully developed when plants are acclimatized to the external environment, and their anatomical features closely resembled those of maternal plants in the wild (Shekhawat and Manokari, 2018). Taken together, at the time of our anatomical analysis, the stem and root tissues of the *in vitro* regenerated plants were fully differentiated and developed.

Although the anatomical features of the stem and root tissues of *in vitro* regenerated and maternal *A. africana* plants were very similar, the anatomical features of the leaves differed somewhat. Unlike the maternal leaf tissue, the *in vitro* regenerated leaf tissue had unorganized cells, less tissue differentiation and development, and very wide intercellular air spaces. These structural abnormalities are not due to somaclonal variations, but rather a stress response by *in vitro* regenerated plants to culture conditions (Shekhawat and Manokari, 2018). As the *in vitro* regenerated plants gradually adapt to the external environment, their tissues differentiate and fully develop (Shekhawat and Manokari, 2018). The observations made in this study indicate that the anatomical assessment was done when the leaf tissue differentiation of *in vitro* regenerated plants was under way and the leaves had not fully adapted to the external environment. In a similar context, Shekhawat and Manokari (2018) also observed that while field plants possessed well-defined and perfectly arranged cells in leaf tissues of *C. indica*, the leaf tissues of *in vitro* plantlets possessed cells that were unorganized. Further, in agreement with observations by Shekhawat and Manokari (2018), the mesophyll cells were poorly developed in our *in vitro* plants, yet fully differentiated and well-developed in *A. africana* mother plants. When *in vitro* regenerated plants are exposed long enough to the field, the leaf mesophyll progresses to differentiate into palisade and spongy parenchyma cells and fully adapts to photosynthesis (Abd El-Zaher, 2008; Batagin-Piotto et al., 2012; Shekhawat and Manokari, 2018). This indicates that the *in vitro* regenerated *A. africana* leaf tissues were not yet fully differentiated and adapted. The non-differentiation of the mesophyll tissues into spongy and palisade layers is attributed to low light intensity in culture rooms and, hence, low photosynthetic rate (Shekhawat and Manokari, 2018). Larger intercellular air spaces in the mesophyll tissues have also been observed in other *in vitro* regenerated plant species, including *C. indica* (Shekhawat and Manokari, 2018) and *Bactris gasipaes* (Batagin-Piotto et al., 2012). Air humidity is very high (about 100%) in *in vitro* culture environments, and *in vitro* plant tissues respond by increasing intercellular air spaces and parenchymatic cell size (Paunescu, 2008). As leaves become more adapted in the field and with differentiation of the mesophyll tissues, air spaces narrow and gradually become normal (Shekhawat and Manokari, 2018). The features noted through cross sections of the tissues of *A. africana* in our study, such as the presence of secretory ducts, uniseriate epidermises, and five (5) vascular bundles (three prominent ones) in leaves, have been previously reported (Mabel et al., 2014; Ekeke and Mensah, 2015).

## Conclusion

Full strength MS medium fortified with 1.0 mg/l BA regenerated the highest number of shoots (13.0 ± 0.424) per nodal explant of *A. africana*, and MS medium supplemented with 0.1 mg/l NAA produced the maximum roots (13.10 ± 0.873) with good shoot length (136.35 ± 4.316 mm). The *in vitro* regenerated *A. africana* plants, upon acclimatization, had a survival rate of up to 95.7%. Furthermore, through our anatomical, physiological, and phytochemical studies, we established that, the *in vitro* regenerated *A. africana* plants closely resembled the maternal plants in most measured parameters. The presence of secretory ducts in the stem tissues of *A. africana*, as well as the detailed anatomical features of its roots, are reported for the first time in our study. The present study provides an efficient repeatable protocol for the *in vitro* regeneration of *A. africana*, which could be employed for large-scale multiplication of the plant in a short time and, thus, greatly contribute toward its domestication and germplasm preservation. To the best of our knowledge, this is the first study not only on *A. africana in vitro* regeneration from nodal segments but also on its *in vitro* propagation as a whole.

## Data Availability Statement

The raw data supporting the conclusions of this article will be made available by the authors, without undue reservation.

## Author Contributions

DO conceived the research idea, designed the experimental plan, participated in every stage and all parts of the research work, did the statistical analyses, and wrote the manuscript. RK participated in the anatomical and SPAD experiments. Y-GK and YC performed the FT-NIR analysis. SY did the histological analysis. ER collected the SPAD experimental data. RG collected the plant materials and wrote the manuscript. FO read and improved the manuscript. YK provided the technical guidance, supervised the whole research work, read and improved the manuscript. All authors read and approved the final manuscript.

## Conflict of Interest

The authors declare that the research was conducted in the absence of any commercial or financial relationships that could be construed as a potential conflict of interest.

## Publisher’s Note

All claims expressed in this article are solely those of the authors and do not necessarily represent those of their affiliated organizations, or those of the publisher, the editors and the reviewers. Any product that may be evaluated in this article, or claim that may be made by its manufacturer, is not guaranteed or endorsed by the publisher.
