# Supplementary material for: An in vitro Propagation of Aspilia africana (Pers.) C. D. Adams, and Evaluation of Its Anatomy and Physiology of Acclimatized Plants
Source: Front Plant Sci. 2021 Jul 29;12:704896. doi: 10.3389/fpls.2021.704896 (PMC8358661; doi:10.3389/fpls.2021.704896)
Supplement: Supplementary file 1 [file Table_1.docx]

| **Plant tissue culture medium** | **Initial shoot length (mm)** | **Final shoot length (mm)** | **Increase in shoot length (%)** | **Initial leaf number** | **Final leaf number** | **Increase in leaf number (%)** | **Initial fresh weight (g)** | **Final fresh weight (g)** | **Increase in fresh weight (%)** | **Overall increase in growth index** |
| --- | --- | --- | --- | --- | --- | --- | --- | --- | --- | --- |
| LS | 32.0 ± 1.453 | 97.50 ± 3.807 | 205.8 ± 4.298^b^ | 3.0 ± 0.333 | 11.0 ± 1.000 | 275.0 ± 8.333^a^ | 0.200 ± 0.0176 | 2.713 ± 0.2680 | 1262.2 ± 44.798^b^ | 581.0 ± 341.15^a^ |
| WPM | 28.0 ± 2.595 | 107.6 ± 7.242 | 292.1 ± 11.932^a^ | 2.5 ± 0.269 | 10.2 ± 0.867 | 316.7 ± 17.213^a^ | 0.138 ± 0.0161 | 2.473 ± 0.2625 | 1714.8 ± 47.105^a^ | 777.8 ± 468.51^a^ |
| DJ | 29.5 ± 2.391 | 99.30 ± 7.140 | 239.4 ± 5.140^ab^ | 2.8 ± 0.291 | 11.3 ± 0.700 | 325.0 ± 31.842^a^ | 0.168 ± 0.0223 | 2.640 ± 0.1495 | 1621.6 ± 130.62^a^ | 728.7 ± 447.15^a^ |
| MS | 31.7 ± 2.463 | 119.4 ± 9.286 | 280.7 ± 13.902^a^ | 2.5 ± 0.269 | 10.0 ± 0.730 | 315.0 ± 25.874^a^ | 0.207 ± 0.0147 | 3.563 ± 0.3172 | 1619.7 ± 85.220^a^ | 741.8 ± 439.14^a^ |
| NM | 30.0 ± 2.662 | 84.70 ± 7.443 | 182.0 ± 13.164^b^ | 3.3 ± 0.300 | 8.3 ± 0.517 | 166.7 ± 22.361^b^ | 0.203 ± 0.0194 | 2.473 ± 0.2615 | 1110.0 ± 49.224^b^ | 558.9 ± 354.85^a^ |
| QL | 30.0 ± 2.662 | 100.4 ± 11.308 | 261.0 ± 10.377^a^ | 4.0 ± 0.000 | 10.0 ± 0.789 | 150.0 ± 19.720^b^ | 0.178 ± 0.0315 | 2.345 ±0.3504 | 1265.8 ± 57.348^b^ | 486.2 ± 311.91^a^ |

**Supplementary materials**

**Table 1: Plant tissue culture media effects on shoot growth of *A. africana* plants from shoot tip explants**

Means (± standard error) within a column followed by same letter are not significantly different by Tukey’ s test and p= 0.05; LS- Linsmaier and Skoog, WPM- Woody Plant Medium, DJ- De Greef and Jacobs, MS- Murashige and Skoog, NM- Nitsch medium, QL- Quoirin and Lepoivre.

**Table 2: Effects of different cytokinins on *in vitro* shoot regeneration of *A. africana* from nodal explants**

| **Cytokinin (mg/l)** | | **Percentage regeneration** | **Number of shoots** |
| --- | --- | --- | --- |
| BA | 0.1 | 100 ± 0.00^a^ | 10.75 ± 0.486^b^ |
|  | 0.5 | 100 ± 0.00^a^ | 6.5 ± 0.267^d^ |
|  | 1.0 | 100 ± 0.00^a^ | 13.0 ± 0.423^a^ |
|  | 1.5 | 100 ± 0.00^a^ | 8.75 ± 0.584^c^ |
|  | 2.0 | 100 ± 0.00^a^ | 5.0 ± 0.397^e^ |
| TDZ | 0.1 | 100 ± 0.00^a^ | 2.5 ± 0.136^gh^ |
|  | 0.5 | 100 ± 0.00^a^ | 2.25 ± 0.099^gh^ |
|  | 1.0 | 100 ± 0.00^a^ | 2.3 ± 0.105^gh^ |
|  | 1.5 | 100 ± 0.00 ^a^ | 2.5 ± 0.154^gh^ |
|  | 2.0 | 100 ± 0.00^a^ | 2.45 ± 0.135^gh^ |
| Kn | 0.1 | 100 ± 0.00^a^ | 3.1 ± 0.250^fg^ |
|  | 0.5 | 100 ± 0.00^a^ | 2.25 ± 0.099^gh^ |
|  | 1.0 | 100 ± 0.00^a^ | 2.65 ± 0.196^fg^ |
|  | 1.5 | 100 ± 0.00^a^ | 3.3 ± 0.206^fg^ |
|  | 2.0 | 100 ± 0.00^a^ | 3.35 ± 0.233^fg^ |
| 2iP | 0.1 | 100 ± 0.00^a^ | 2.25 ± 0.099^gh^ |
|  | 0.5 | 100 ± 0.00^a^ | 2.45 ± 0.114^gh^ |
|  | 1.0 | 100 ± 0.00^a^ | 2.25 ± 0.099^gh^ |
|  | 1.5 | 100 ± 0.00^a^ | 2.45 ± 0.135^gh^ |
|  | 2.0 | 100 ± 0.00^a^ | 2.25 ± 0.099^gh^ |
| Z | 0.1 | 100 ± 0.00^a^ | 3.85 ± 0.254^ef^ |
|  | 0.5 | 100 ± 0.00^a^ | 2.8 ± 0.186^fgh^ |
|  | 1.0 | 100 ± 0.00^a^ | 2.4 ± 0.152^gh^ |
|  | 1.5 | 100 ± 0.00^a^ | 3.2 ± 0.200^fg^ |
|  | 2.0 | 100 ± 0.00^a^ | 2.9 ± 0.228^fgh^ |
| MS (Control) | | 80 ± 5.41^b^ | 1.8 ± 0.138^h^ |

Means (± standard error) within a column followed by same letter are not significantly different by Tukey’ s test and p= 0.05; BA- 6-Benzylaminopurine, TDZ- Thidiazuron, Kn- Kinetin, 2iP- Isopentenyl adenine, Z- Zeatin.

Means (± standard error) within a column followed by same letter are not significantly different by Tukey’ s test and p= 0.05; IAA- indole-3-acetic acid, IBA- indole-3-butyric acid, NAA- naphthaleneacetic acid, MS- Murashige and Skoog plant tissue culture medium.

| **Auxin (mg/l)** | | **Percentage rooting** | **Number of roots** | **Root length (mm)** |
| --- | --- | --- | --- | --- |
| IAA | 0.1 | 60 ± 12.47^b^ | 3.3 ± 0.729^cd^ | 23.25 ± 3.718^e^ |
|  | 0.25 | 80 ± 11.06^ab^ | 4.45 ± 0.667^bc^ | 26.4 ± 2.388^e^ |
|  | 0.5 | 80 ± 8.16^ab^ | 3.55 ± 0.540^c^ | 28.81 ± 2.759^e^ |
|  | 0.75 | 80 ± 8.16^ab^ | 3.75 ± 0.475^c^ | 26.5 ± 2.652^e^ |
|  | 1.0 | 55 ± 13.84^b^ | 1.15 ± 0.264^d^ | 16.08 ± 1.940^e^ |
| IBA | 0.1 | 100 ± 0.00^a^ | 4.35 ± 0.372^cd^ | 68.45 ± 3.221^c^ |
|  | 0.25 | 100 ± 0.00^a^ | 12.55 ± 0.634^a^ | 89.6 ± 3.579^b^ |
|  | 0.5 | 100 ± 0.00^a^ | 8.05 ± 0.875^b^ | 55.8 ± 1.923^c^ |
|  | 0.75 | 100 ± 0.00 ^a^ | 12.1 ± 0.602^a^ | 61.5 ± 2.529^c^ |
|  | 1.0 | 90 ± 6.67^ab^ | 11.5 ± 1.111^a^ | 37.83 ± 2.456^de^ |
| NAA | 0.1 | 100 ± 0.00^a^ | 13.1 ± 0.873^a^ | 136.35 ± 4.316^a^ |
|  | 0.25 | 90 ± 6.67^ab^ | 5.7 ± 0.548^bc^ | 51.65 ± 1.948^cd^ |
|  | 0.5 | 100 ± 0.00^a^ | 11.05 ± 0.709^ab^ | 43.95 ± 2.543^cd^ |
|  | 0.75 | 100 ± 0.00^a^ | 11.5 ± 0.473^a^ | 43.55 ± 2.323^cd^ |
|  | 1.0 | 100 ± 0.00^a^ | 7.6 ± 0.845^b^ | 51.9 ± 1.583^c^ |
| MS (Control) | | 80 ± 11.06^ab^ | 4.05 ± 0.604^cd^ | 42.29 ± 2.601^de^ |

**Table 3: Effects of auxins on *in vitro* rooting of regenerated *A. africana* shoots from nodal explants**

Means (± standard error) within a column followed by same letter are not significantly different by Tukey’ s test and p= 0.05; IAA- indole-3-acetic acid, IBA- indole-3-butyric acid, NAA- naphthaleneacetic acid.
